# Alcohol Counter-Advertising and the Media: A Review of Recent Research

**Published:** 2002

**Authors:** Gina Agostinelli, Joel W. Grube

**Affiliations:** Gina Agostinelli, Ph.D., is a research scientist, and Joel W. Grube, Ph.D., is a senior research scientist and associate director at the Prevention Research Center, Berkeley, California

**Keywords:** counter-advertising, alcohol or other drug (AOD) product advertising, AOD advertising impact, warning label, public service announcement, mass media prevention approach, behavioral change, survey of research

## Abstract

Counter-advertising commonly is used to balance the effects that alcohol advertising may have on alcohol consumption and alcohol-related problems. Such measures can take the form of print or broadcast advertisements (e.g., public service announcements [PSAs]) as well as product warning labels. The effectiveness of both types of counter-advertising is reviewed using the Elaboration Likelihood Model as a theoretical framework. For print and broadcast counter-advertisements, such factors as their emotional appeal and the credibility of the source, as well as audience factors, can influence their effectiveness. Further, brewer-sponsored counter-advertisements are evaluated and received differently than are the more conventional PSA counter-advertisements. For warning labels, both the content and design of the label influence their effectiveness, as do audience factors. The effectiveness of those labels is evaluated in terms of the extent to which they impact cognitive and affective processes as well as drinking behavior.

Widespread concern exists among policymakers and the public about the potential effects of alcohol advertising on alcohol consumption and problems, especially among children and adolescents. It is especially important to counter the potential effects of advertising on young people because these age groups may be more susceptible to those effects. Children are less able to discriminate between advertising and other media content and are less critical of commercial messages than are adults (Atkin 1995). Moreover, recent studies of children and adolescents (e.g., Casswell and Zhang 1998; Grube and Wallack 1994; Wyllie et al. 1998) have shown that attention to and liking of alcohol advertising are related to (1) greater knowledge about alcohol slogans and beer brands, (2) more favorable beliefs about drinking, (3) increased intentions to drink as an adult, and (4) increased drinking. Similarly, it may be important to counter the potential effects of alcohol advertising on young adults, and especially college students, who frequently are at risk for heavy and problematic drinking (Wechsler et al. 2000).

A recent national survey indicates that 67 percent of adults in the United States support banning liquor advertisements on television and 61 percent favor banning beer and wine advertisements in this medium (Wagenaar et al. 2000). Similarly, public health advocates routinely call for the strict regulation or even elimination of alcohol advertising, and initiatives at the community level frequently focus on reducing local alcohol advertising. In part, concerns about alcohol advertising result from its pervasiveness. In 1999, the alcoholic beverage industry spent $1.24 billion on alcohol advertising (Center for Science in the Public Interest 2001). Most of these expenditures ($796.3 million) were concentrated on television and radio commercials; among beverage types, beer advertising accounted for the majority of the spending ($799.7 million). Consistent with these data, studies document that alcohol advertising, and particularly beer advertising, is a relatively frequent occurrence on television, especially in sports programming. For example, approximately two alcohol advertisements appear in each hour of major professional sports programming, compared with approximately one alcohol advertisement in every 4 hours of entertainment programming (Grube 1993, 1995; Madden and Grube 1994).

To address and counteract the pervasiveness of alcohol advertising, policymakers can take several approaches. In addition to the restrictions on alcohol advertising discussed above, counter-advertising—the presentation of factual information and persuasive messages through the media—is such an approach. It primarily takes two forms: (1) broadcast (e.g., television and radio), outdoor (e.g., billboard), and print counter-advertisements, and (2) product warning labels. This article reviews the effectiveness of these two general types of counter-advertising in changing drinking-related beliefs, intentions, and behaviors. First, however, it presents a useful model for understanding and assessing media persuasion effects and the relative endurance and direction of such effects—the Elaboration Likelihood Model (ELM) (Petty and Cacioppo 1986*a*, b; Petty and Priester 1994).

## The Elaboration Likelihood Model

The ELM distinguishes two routes through which counter-advertising may persuade target audiences to change their attitudes and behaviors—a central route and a peripheral route. The central route involves a high level of issue-relevant thinking. This means that message recipients are likely to carefully attend to the content of the message; scrutinize and elaborate upon this content in light of their own knowledge; decide on the merits of its arguments; and consequently derive an overall evaluation, or attitude, toward that message. Through this effortful reasoning process, the recipients integrate the provided information into their own belief structures, which then may result in attitude change. In contrast, the peripheral route involves a less effortful reasoning process that does not rely on scrutinizing the content and merits of the message. Here, attitudes are formed based on relatively simple cues without issue-relevant thinking. For example, the very nature of a communicator being highly credible and/or attractive may be enough to automatically lead the message recipient to accept the recommendation without giving the provided arguments any serious thought.

The ELM posits that attitude change mediated through the more effortful central route will be longer lasting, more resistant, and more predictive of behavior than change mediated through the peripheral route. According to this model, the goal of prevention experts in designing effective counter-advertisements and warning labels therefore should be to induce change through the central route. The model also specifies that audience members must be both able and motivated to take the more lasting and effortful central route. In situations where the audience may only be moderately interested in a topic, factors that otherwise might act as peripheral cues (e.g., source credibility and source attractiveness) can also affect whether audience members engage in the more effortful central route. For example, college students, particularly fraternity members, who may be only moderately interested in messages about abstinence or responsible drinking might be more likely to deeply process a message delivered by a favorite athlete. Yet even central processing of a message does not guarantee that the message will result in attitude change in the intended direction. Once people process information through the central route, the effect of the message depends upon how each person responds to it. For example, a heavy drinker could react defensively with his or her own set of biased thoughts to the provided counter-advertisement, doubting the merits of the information provided and becoming even more convinced of the legitimacy of his or her initial heavy drinking stance. In such a case, the persuasion attempt would thus backfire and further polarize an already pro-drinking attitude.

## Effectiveness of Broadcast and Print Counter-Advertising

Counter-advertisements that recommend responsible alcohol use generally are conveyed to the public through television, radio, outdoor, or print media. These messages can be either produced by government agencies or community action groups, or they can be industry-sponsored. The broadcast of counter-advertisements also can be either donated (e.g., public service announcements [PSAs]) or purchased (e.g., social marketing). The issue of drinking and driving has been a primary target of counter-advertising campaigns.

Following a rigorous analysis of the contents of drinking-and-driving PSAs, Slater (1999*a*) identified the most common strategy in such campaigns as the informational/testimonial approach, which provides basic facts or simply exhorts appropriate behavior. The information may be delivered as a testimonial by a celebrity or a person on the street, or in a more educational format as simple information to be learned. This approach does not employ strong elements of the other four common strategies identified by Slater (1999*a*). These other strategies are those that (1) model appropriate behavior (e.g., giving up car keys after drinking), (2) employ positive appeals (e.g., depicting enjoyable social situations without drinking), (3) evoke alcohol-related fear (e.g., of accidental death), and (4) evoke empathy (e.g., for victims of drunk drivers).

The most common informational/testimonial approach assumes that providing information will increase audience knowledge and awareness of the drunk-driving issue and eventually impact the targeted behavior. When analyzed from the perspective of the ELM, however, such messages are not always designed in a way that optimizes their long-term effectiveness. For example, although informational/testimonial PSAs provide relevant information that people can integrate into their belief systems, these PSAs are not necessarily appealing in their design and therefore are less likely to interest and motivate people to process them more carefully through the central route.

Recently, Austin and colleagues (1999) compared the effectiveness of antidrinking PSAs to the alcohol advertisements that they were supposed to counter. In that study, college students rated both PSAs (e.g., “Friends don’t let friends drive drunk”) and alcohol advertisements (e.g., for Bud Lite) on dimensions related to whether they would attract interest and motivate people to process them. In general, the college students rated the PSAs as less enjoyable and appealing but also as more realistic, honest, and effective. The investigators also examined to what extent the frequency with which college students consumed alcohol influenced their ratings of the alcohol advertisements and PSAs. This analysis found that even though alcohol advertisements were generally rated as less effective than the PSAs, the more frequently college students reportedly consumed alcohol, the more favorably they responded to the alcohol advertisements. For example, more frequent^1^ drinkers rated the alcohol advertisements as more effective, identified with their portrayals more, and rated those portrayals as more desirable compared with others. More frequent drinkers also rated the PSAs as less effective than did other students. The investigators concluded that an overemphasis on logic-based realistic and honest appeals in conventional PSAs, at the expense of the more emotion-based appeal of alcohol advertisements, can compromise the PSAs’ effectiveness.

Future research still must determine, however, whether the appealing features of the alcohol advertisements translate, over time, into a deep central processing of such advertisements with an enduring attitude and behavior change, or whether these features contribute only to peripheral-route processing. The influence of a person’s drinking level in this process should also be examined. Designers of alcohol counter-advertisements should then employ those advertising features that best promote central processing for heavy drinkers, light drinkers, and nondrinkers alike.

Another set of analyses of alcohol counter-advertising was conducted after a 1988 Surgeon General’s workshop on drunk driving called for mass communication campaigns directed at the prevention of alcohol-related traffic deaths. To examine the impact of such campaigns, Dejong and Atkin (1995) analyzed the contents of PSAs aired nationally between 1987 and 1992. This analysis identified two dominant types of PSAs in the campaign that correspond to classic peripheral factors of influence posited by the ELM:

- Celebrity endorsements with a “talking head” format (e.g., Magic Johnson stating that a designated driver is the most valued player)
- Emotional appeals attempting to evoke fear, anger, and empathy (e.g., PSAs by Mothers Against Drunk Driving [MADD] expressing both anger at drunk drivers and sympathy for their innocent victims).

The main objective of these PSAs was to encourage the adoption of more responsible drinking-related behaviors, such as using designated drivers and intervening to prevent alcohol-impaired people from driving. Indeed, after the campaign, Gallup surveys offered strong evidence of a sharp drop in the number of impaired drivers on the road (Dejong and Hingson 1998). In addition, a remarkable decline in the number of U.S. alcohol-related traffic deaths occurred between 1982 and 1996 (Dejong and Hingson 1998).

To some extent, these decreases might be attributable to the PSA campaign. Yet, as Dejong and Hingson (1998) warn, determining the unique contribution of any single initiative to such favorable outcomes is fraught with methodological difficulties. Several simultaneous legal and programmatic initiatives within the broader drunk-driving campaign (e.g., sobriety checkpoints, increased minimum legal drinking age, and responsible beverage service) as well as other shifts in regulations affecting risky driving behaviors (i.e., speeding laws) also contributed to the observed effects.

Accordingly, future research must determine whether, and by what processes, the classic celebrity endorsement and emotional-appeal PSAs uniquely contribute to attitude and behavior change. For example, celebrity endorsements could promote a shallower peripheral processing of a PSA, with a more temporary attitude and behavior change, simply because the messages of well-liked and credible celebrities may go unchallenged without ever motivating viewers to think deeply about the message content. Conversely, celebrities could promote a deeper central processing and more enduring attitude change by attracting the attention of those viewers who initially were only mildly interested and motivate those viewers enough to carefully process and accept their messages.

### Brewery-Sponsored Counter-Advertisements

Some counter-advertisements also have been sponsored by beer brewers, and researchers have compared the reactions, particularly of young viewers (i.e., ages 16–22 years) to these brewer-sponsored messages with more conventional PSA counter-advertisements (Atkin et al. 1992, 1994). The brewer-sponsored counter-advertisements studied were from Anheuser-Busch’s “Know when to say when” and Coors’ “Now, not now” campaigns, both of which were purportedly created to promote safe and responsible drinking. According to the researchers, their content reflects a hybrid of commercial, public relations, and public service persuasion strategies. Thus, these brewer-sponsored counter-advertisements tend to be “soft sell” versions of traditional PSAs. In contrast, nonindustry PSAs (e.g., those sponsored by MADD or the Ad Council) tend to be straightforward fear appeals that have more explicit guidelines and are generally slower-paced and less entertaining.

Study participants rated the brewer-sponsored counter-advertisements as less informative, believable, on-target, and effective than the conventional PSAs. Furthermore, when asked to rank the motives for the brewer-sponsored counter-advertisements, the study participants rated the prevention of drunk driving only third, behind improvement of the company’s image and selling its beer. Thus, these young viewers received the brewer-sponsored PSAs with skepticism.

The young study participants also viewed the brewer-sponsored counter-advertisements as permitting liberal alcohol consumption, even in risky situations. This latter effect may result from the use of strategic ambiguity, which sends an unclear message about how much to limit one’s drinking (Atkin et al. 1994). This means that the messages sanction an acceptable level of drinking but leave it to the viewer to decide what that level is. For example, in the “Know when to say when” counter-advertisements, the “when” and how to “know when” are never defined. Even with less ambiguous messages (e.g., a NASCAR Budweiser driver stating “Please, don’t drink and drive”) or with messages directly modeling choosing a designated driver, other cues in the advertisements may create ambiguity or even serve as peripheral cues in promoting pro-drinking attitudes. Such cues may include Budweiser logos promoting beer sales or people at a party enjoying alcoholic beverages.

Another problem associated with brewery-sponsored counter-advertising is that by the very act of airing a communication that promotes restricted alcohol use, the alcohol industry seemingly argues against its own interest and paradoxically may increase its credibility and persuasive power through peripheral processing mechanisms (Petty and Cacioppo 1981). Indeed, despite the evidence for some skepticism toward brewer-sponsored PSAs, youth rated the beer industry as respectable, responsible, and caring after viewing the industry-sponsored counter-advertisements (Atkin et al. 1994). The researchers concluded that, taken together, these findings suggest an unfortunate effect of brewer-sponsored counter-advertisements when compared to more conventional PSAs—that despite their initial intent, for youth, the brewer-sponsored advertisements may justify drinking in risky situations and promote alcohol sales more generally (Atkin et al. 1994).

### The Influence of Audience Factors

Audience factors also can affect the extent to which counter-advertising leads to attitude and behavior change. For example, videotaped alcohol counter-advertisements are most effective when the communicator is of the same gender as the viewer, and they have a greater influence on lighter than on heavier drinkers (Bochner 1994).^2^ Consistent with the previously discussed finding that heavy drinkers rated PSAs as less effective than did lighter drinkers (Austin et al. 1999), this latter observation suggests that heavier or problematic drinkers may be particularly resistant to counter-advertising (Dejong and Atkin 1995). For this reason, Isaac (1995) recommended that drunk-driving media campaigns not target at-risk drinkers but rather urge the intervention by friends who are more likely to be respected and be listened to by resistant drinkers.

Nonetheless, even young people with a “sensation-seeking” personality^3^ who are prone to alcohol and other drug abuse can be directly influenced with properly designed counter-advertising. For example, such people are more likely to call a hot line mentioned in an anti-drug PSA when the PSA is high in sensation value (i.e., fast-paced, upbeat, and suspenseful). Conversely, young people with a less sensation-seeking personality are more responsive to PSAs with lower sensation value (Donohew et al. 1994).

The optimal design of counter-advertisements for changing problematic behavior also may depend upon what stage a person has reached in being able to recognize his or her own behavior as problematic (Slater 1999*b*). For example, people who do not yet recognize that their drinking and driving is problematic and have a limited motivation to think about the issue likely will ignore a purely logic-based message unless it has appealing features (e.g., is dramatic enough to attract attention or is communicated by a well-liked celebrity). For people who are already motivated to change, however, messages modeling the desired behaviors (e.g., how to tactfully refuse drinks at a party) could facilitate the translation of the drinkers’ intentions into the desired behaviors by providing specific information on how to enact those behaviors.

Taken together, the research on message, source, and audience factors indicates that part of the success of counter-advertising will depend on how these factors interact. Based on the ELM, which predicts that central route processing leads to more durable attitude and behavior change, it appears critical that counter-advertisements include strong logical arguments for audience members to integrate into their belief structures. Yet, for those audience members who are not motivated to attend to these messages, peripheral factors (e.g., appeal, celebrity endorsements, and sensational content) should be employed as well to maximize the likelihood that all audience members are attracted to and process the rich message content. Indeed, consistent with the ELM, Slater’s (1999*b*) analysis suggests that counter-advertisements should employ both peripheral factors to ensure that the advertisements are appealing and motivate the audience to process them and strong logical information that can be integrated into belief systems to ensure a lasting change.

## Effectiveness of Warning Labels

The second form of counter-advertising is alcoholic beverage warning labels and posters. Health warning label legislation was implemented in the United States in 1989. This legislation requires all alcoholic beverage containers to bear a government warning of the risks associated with consuming alcohol while pregnant, driving a car, or operating machinery. Similarly, since the 1980s several States have mandated the display of health warning posters at places where alcohol is sold.

Of primary importance in studying the effectiveness of warning labels is the most basic research question—do people even notice the labels? Several studies have explored message-design factors that influence whether the labels are noticed (for reviews, see Andrews 1995; MacKinnon 1995). In three separate experiments, Laughery and colleagues (1993) measured how quickly people could locate warning labels on alcoholic beverage containers. The investigators concluded that the typical alcohol warning labels are not particularly noticeable because they blend in with their backgrounds. Several factors, such as clutter on the labels themselves, their vertical placement on the container, and placement other than on the front of the container, make the warning less noticeable. Further, the use of pictorials, icons, and color improve the labels’ noticeability. From the perspective of the ELM, these findings indicate that the first critical step in designing effective warning labels should be to ensure that audience members can notice and thus further process the labels.

Once a warning label is noticed, its content becomes of paramount importance. To identify the factors influencing the effectiveness of alcohol warning labels, MacKinnon and colleagues (1994) systematically varied several features of warning labels, such as their length, the use of qualifier words (e.g., *may* cause cancer), and the specific risks mentioned (e.g., birth defects, health risks, or cancer). To determine whether certain bottle warning labels would be more successful in leading people to avoid those bottles, participants were asked to imagine that they were in a supermarket and had to choose between two different bottles. This test was repeated with several pairs of bottles, with bottles in each pair displaying one of the possible warning labels and a blank label. When participants chose the bottle with the blank label within each pair instead of the bottle with the experimental warning label, this signaled that the experimental warning label effectively led people to avoid or not choose the bottle on which it was displayed. For both college and high school students, the study had the following results:

- The specific risks mentioned on the label were more important in determining choices than was the label length.
- Bottles with labels containing qualifier words were avoided less than were bottles without such words.
- Whiskey bottles with warning labels were avoided more than were beer bottles with warning labels.
- Alternative warnings containing the words “poison” and “cancer” elicited more avoidance than did the currently used Surgeon General’s alcohol warning label.

Together, these findings indicate that research participants under these experimental conditions noticed the content of the warning labels and processed that content at some minimal level. At the same time, the results point to a less thought-engaging and more peripheral process potentially underlying effective counter-advertising. That is, the alternative warning labels presumably worked by producing a visceral avoidance response. The mere association of a bottled product with negative words (e.g., “poison”) may have effectively generated an automatic repulsive avoidance of the product, consistent with classical conditioning^4^ mechanisms. It would be of interest to determine in future research whether the obtained findings would also generalize to other young adults not in college as well as to older adults.

Whereas this experimental research examined the influence of the content of an alcohol warning label on its ability to evoke avoidance responses, later research has evaluated how deeply warning labels are being processed and whether they influence drinking behavior. Various studies document that the general public’s awareness of warning labels and posters is high (Hilton 1993; International Center for Alcohol Policies [ICAP] 1997; Kaskutas and Greenfield 1997; MacKinnon 1995). A more recent study focused on 10th and 12th grade students’ responses to warning labels during the first 5 years that those labels were required (MacKinnon et al. 2000). Despite the fact that it is illegal for adolescents to drink alcohol, experimentation with alcohol typically begins in adolescence, and many attitudes regarding alcohol use are established during this period. It is therefore instructive to study how this population processes these labels. This study found that the initial positive effects of the warning labels on adolescents’ awareness of, exposure to, and recognition of these warnings were beginning to level off over the course of the study. Taken together, the accumulated evidence suggests that the warning labels are being noticed and their content is remembered. The findings are less clear, however, as to what people are learning or comprehending from these labels, how different people react to these labels, and whether people’s behavior is affected as intended.

For example, evidence concerning how deeply the information content of the warning labels is processed and to what extent readers comprehend and accept the risks communicated (e.g., pregnancy complications and drunk-driving risks) is mixed. Some researchers have argued that a “ceiling effect” exists—that warning labels are ineffective in teaching the general public anything new about the targeted drinking-related risks because the readers already know about these risks (Hilton 1993; ICAP 1997; MacKinnon 1995). Furthermore, the aforementioned study on warning label effects in adolescents found no beneficial change in terms of alcohol-related beliefs, consumption, or driving after drinking that was attributable to the labels (MacKinnon et al. 2000). Other studies also found evidence that alcohol warning labels do not affect drinking behavior (Andrews 1995; Hilton 1993; MacKinnon 1995).

Some more recent evidence suggests, however, that warning labels may have delayed behavioral effects. Specifically, Greenfield (1997) found that a person’s ability to recall the drinking-and-driving message on alcohol warning labels predicted the self-reported likelihood to limit both driving after drinking and drinking when planning on driving. Further, Hankin and colleagues (1993) documented that in a traditionally hard-to-reach population of pregnant, inner-city African American women, alcohol consumption among low-risk drinkers declined after the introduction of warning labels.

Experimental research that varies exposure to alcohol warnings to address the question of how these warnings affect people similarly has found mixed results for their effectiveness. MacKinnon’s (1995) review raises the potential for “overwarning” effects—that people become overly accustomed to warnings and, as a result, ignore them or, worse yet, react to them unfavorably. A study by Snyder and Blood (1992) found some evidence for such an effect. In that study, college student drinkers exposed to the Surgeon General’s alcohol warning in a printed alcohol advertisement perceived greater benefits from drinking than did college student drinkers exposed to the same advertisement without the warning. In addition, male drinkers exposed to the warning expressed greater intentions to drink than did those exposed to the same advertisement without the warning. Other researchers failed to replicate these effects in two separate experiments, however, leading them to conclude that the earlier observed effect was based on inappropriate statistical comparisons and confounding factors (MacKinnon and Lapin 1998). Slater and Domenech (1995) also have pointed to the weak nature of the warning employed by Snyder and Blood (1992), which may have influenced the results.

From the perspective of the ELM, the effect of a warning message will depend upon how its audience reacts to it. For a warning to effectively counter an alcohol advertisement, it must receive favorable reactions and generate supportive thoughts. For example, Slater and Domenech (1995) demonstrated that repeated exposure to alcohol warnings that were embedded within beer advertisements elicits negative beliefs to counter-argue those advertisements and leads viewers to be less confident about the benefits of beer drinking. Further, Andrews (1995) indicated that one’s own conscious thoughts elicited in response to the warning labels (i.e., one’s cognitive responses) mediate approximately three-quarters of the effects that warning labels have on how favorably the labels are evaluated. Thus, self-generated thoughts in response to reading warning labels are important intermediate variables in determining whether the warning labels will be persuasive. Together, this research indicates that investigators and policymakers must understand how people cognitively react to alcohol warnings so as to design warnings that produce the intended antidrinking attitudes or at least erode the confidence of pro-drinking beliefs.

Several audience factors also predict the depth to which people process alcohol warning labels and whether they react favorably or unfavorably to them. For example, researchers found that the ability to recall container warning label messages is highest among younger respondents, heavier drinkers, and purchasers of alcohol (Kaskutas and Greenfield 1997). This finding suggests that the messages do indeed reach the target groups. Yet, although heavy drinkers are aware of drinking risks, they also discount warning labels and perceive them less favorably and as less believable than do light drinkers (Andrews 1995; Andrews et al. 1991).

Similarly, in the study of pregnant, inner-city African American women, only the lighter drinkers who were less at risk of having children with alcohol-related birth defects heeded warning labels (Hankin et al. 1993). Finally, younger, pregnant, inner-city African American women were more aware of and more likely to believe the birth defects warning and limit their drinking than were their older peers, despite the fact that the risks for alcohol-related birth defects increase with age (Hankin 1996). These findings indicate that although the people most in need of adopting alcohol warnings are aware of and can recall the information in warning labels, these same people are least likely to accept the warnings.

Taken together, the research on the design and content of warning label factors as well as on audience factors indicates that the effectiveness of warning labels on drinking behavior depends on how these factors initially impact underlying cognitive and affective processes. First, design factors influence whether warning labels are even initially noticed. Second, the specific content of warning labels could influence the labels’ potential for evoking visceral avoidance responses. Third, audience factors predict differential memory for, processing of, and reactions to alcohol warning labels. These audience effects can then modify drinking behavior.

## Conclusion

To dilute the influence of alcohol advertising, broadcast and print counter-advertising and warning labels present factual information and persuasive messages to the public. Some evidence supports the effectiveness of these strategies, although the findings are mixed and typically qualified by message, source, and audience factors. To predict the conditions under which various counter-advertising approaches will work, researchers must understand the processes contributing to or limiting their effectiveness. The ELM provides a useful framework for integrating the emerging findings and for predicting when counter-advertising and warning labels will lead to a more durable attitude change and ultimately affect the behaviors they target.

## Footnotes

1 Definitions of drinking severity (e.g., heavy drinking or moderate drinking) and drinking frequency (e.g., frequent drinker) vary among studies. Therefore, the terms are used in this article as they were in the original studies cited.

2 The definitions associated with different levels of alcohol use varied across Bochner’s (1994) three studies. In study 1, participants were classified as heavy versus light/moderate drinkers, contrasting those who had a drink at least once or twice a week (heavy) to all other participants, including those who never drank (light/moderate). In both studies 2 and 3, participants were classified as moderate/heavy versus light drinkers, contrasting those who had a drink at least once or twice a month (moderate/heavy) to all other participants, including those who never drank (light).

3 Sensation-seeking is characterized by a willingness to take risks for the sake of varied, novel, and intense experiences. It is also associated with alcohol and other drug use (Zuckerman 1994).

4 Classical conditioning allows for an initially neutral stimulus (e.g., new consumer product) to eventually elicit a positive or negative response on its own after repeated associations with an already valued stimulus.
